# Type IV Laryngotracheoesophageal Cleft Associated with Type III Esophageal Atresia in 1p36 Deletions Containing the RERE Gene: Is There a Causal Role for the Genetic Alteration?

**DOI:** 10.1155/2018/4060527

**Published:** 2018-08-29

**Authors:** Gloria Pelizzo, Aurora Puglisi, Maria Lapi, Maria Piccione, Federico Matina, Martina Busè, Giovanni Battista Mura, Giuseppe Re, Valeria Calcaterra

**Affiliations:** ^1^Pediatric Surgery Unit, Pediatric Surgery Unit, Children's Hospital “G. Di Cristina”, ARNAS “Civico-Di Cristina-Benfratelli”, Palermo, Italy; ^2^Pediatric Anesthesiology and Intensive Care Unit, Pediatric Anesthesiology and Intensive Care Unit, Children's Hospital “G. Di Cristina”, ARNAS “Civico-Di Cristina-Benfratelli”, Palermo, Italy; ^3^Department of Sciences for Health Promotion and Mother and Child Care “Giuseppe D'Alessandro”, University of Palermo, Palermo, Italy; ^4^Neonatal Intensive Care Unit, A.O.U.P. “P. Giaccone”, Department of Sciences for Health Promotion and Mother and Child Care “G. D'Alessandro”, Palermo, Italy; ^5^Pediatrics and Adolescentology Unit, Department of Internal Medicine University of Pavia and Fondazione IRCCS Policlinico San Matteo, Pavia, Italy

## Abstract

The causes of embryological developmental anomalies leading to laryngotracheoesophageal clefts (LTECs) are not known, but are proposed to be multifactorial, including genetic and environmental factors. Haploinsufficiency of the RERE gene might contribute to different phenotypes seen in individuals with 1p36 deletions. We describe a neonate of an obese mother, diagnosed with type IV LTEC and type III esophageal atresia (EA), in which a 1p36 deletion including the RERE gene was detected. On the second day of life, a right thoracotomy and extrapleural esophagus atresia repair were attempted. One week later, a right cervical approach was performed to separate the cervical esophagus from the trachea. Three months later, a thoracic termino-terminal anastomosis of the esophagus was performed. An anterior fundoplication was required at 8 months of age due to severe gastroesophageal reflux and failure to thrive. A causal role of 1p36 deletions including the RERE gene in the malformation is proposed. Moreover, additional parental factors must be considered. Future studies are mandatory to elucidate genomic and epigenomic susceptibility factors that underlie these congenital malformations. A multiteam approach is a crucial factor in the successful management of affected patients.

## 1. Introduction

Complete laryngotracheoesophageal clefts (LTECs), types III and IV, are rare congenital anomalies that occur when the primitive foregut fails to separate into the tracheobronchial tree and the esophagus [1]. These malformations are the most challenging to diagnose and manage since they are life-threatening conditions [1–6]. LTECs have been associated with many other congenital anomalies, such as defects of the gastrointestinal and genitourinary tracts and cardiovascular anomalies [7]. The causes of embryological developmental anomalies leading to LTECs are not known, but are proposed to be multifactorial, including genetic and environmental factors, such as maternal risks [4–6, 8].

The arginine-glutamic acid dipeptide repeats gene (RERE (MIM: 605226)) is located in the proximal 1p36 critical region [9, 10]. RERE encodes a widely expressed nuclear receptor coregulator [11, 12] that positively regulates retinoic acid signaling in multiple tissues during embryonic development [13–15]. Data from animal models suggest that haploinsufficiency of RERE might contribute to intellectual disability, developmental delay, structural brain anomalies, vision problems, hearing loss, congenital heart defects, cardiomyopathy, and renal anomalies seen in individuals with 1p36 deletions [16]. However, the exact role that RERE deficiency plays in 1p36 deletion syndrome, and more generally in human disease, remains unclear [9].

We describe a neonate diagnosed with type IV LTEC and type III esophageal atresia in which a 1p36 deletion including the RERE gene was detected. The surgical and anesthesiological decision-making and management of the newborn are detailed. The roles of multifactorial factors in the pathogenesis of the malformation are also discussed.

## 2. Case Presentation

A Caucasian male neonate, weighing 3080 g, was born by cesarean section at the 37th week of pregnancy. Prenatally, at 23 weeks' gestation, microgastria without polyhydramnios was detected and a suspicion of type III esophageal atresia was suggested. Prenatal MRI was not available due to severe maternal obesity. The Apgar scores were 7 and 9 at 1 and 5 minutes, respectively. At birth, clinical examination revealed hypotonia and pathological transmitted sounds from the upper airways without respiratory distress. No dysmorphic features, eye anomalies, vertebral or limb abnormalities, or genitalia malformations were noted.

**Figure 1:**
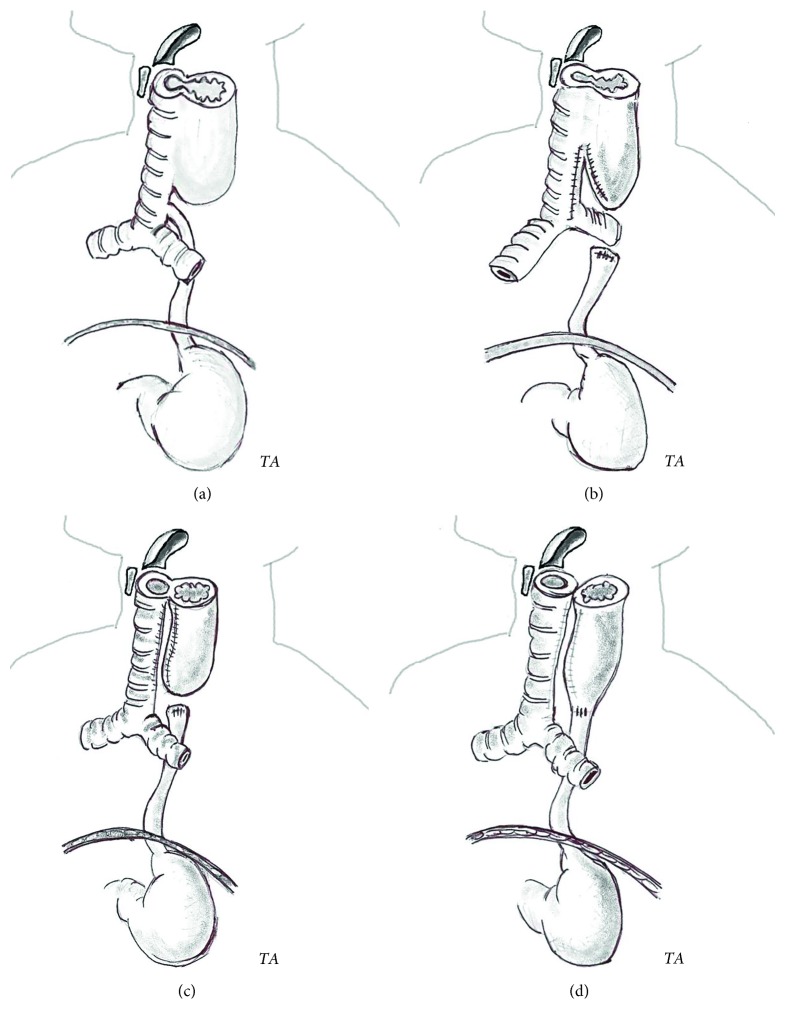
Illustration of the congenital malformation and surgical procedures. (a) Type IV laryngotracheoesophageal clefts (LTEC) and type III esophageal atresia. (b) Step 1: suture of the tracheoesophageal fistula, dissection of the proximal esophageal pouch from the trachea, and separation of the neoesophageal tube from the trachea. (c) Step 2: separation of the cervical esophagus from the trachea. (d) Step 3: thoracic termino-terminal anastomosis of the esophagus (drawn by Dr. Salvatore Amoroso).

A posterior-anterior chest radiograph at 3 h of age confirmed the prenatal suspicion of esophageal atresia. At bronchoscopy, type IV LTEC was diagnosed associated with type III esophageal atresia. Illustration of the malformation is shown in Figure 1(a).

On the second day of life, a right thoracotomy and extrapleural esophagus atresia repair were attempted. Following induction, the patient was intubated with a 3.5 mm uncuffed endotracheal tube. The tracheoesophageal fistula was sutured first. Multiple episodes of desaturation were immediately controlled after the dissection of the proximal esophageal pouch from the trachea. The neoesophageal tube was separated from the trachea using running sutures (Figure 1(b)). A nonresorbable biologic-tissue patch was positioned in between the anterior esophageal wall and the trachea in order to consolidate the tracheal pars membranacea. The proximal and distal pouches were stitched to the right thoracic paravertebral region to obtain progressive elongation. Gastrostomy for feeding was also performed.

One week later, a right cervical approach was performed to separate the cervical esophagus from the trachea (Figure 1(c)). After surgery, the child was gradually weaned off a ventilator, and after 13 days, sufficient respiratory autonomy was obtained. Enteral feeding was started. The postoperative course was characterized by *Klebsiella* septicemia, a thromboembolic event and bronchopneumonia *ab ingestis*. Three months later, the thoracic termino-terminal anastomosis of the esophagus was performed (Figure 1(d)). An anterior fundoplication was required at eight months of age due to severe gastroesophageal reflux and failure to thrive.

Genetic testing and genomic DNA sequencing were performed. No evidence of cystic fibrosis or phenylketonuria was detected. CHD7 sequencing and deletion and duplication analysis did not reveal any pathological variants. Array-CGH analysis documented a 1p36.33 deletion of approximately 50 kb (position from 8.779.410 to 8.830.261), containing the RERE gene. Array-CGH analysis in both parents showed that the rearrangement was of paternal origin. The child exhibited the associated anomalies, unilateral hypoplastic and ptotic right kidney. No pathological neurological signs or symptoms were revealed. The proband's father had a normal phenotype but was severely obese.

## 3. Discussion

To the best of our knowledge, this is the first report on a 1p36 deletion containing the RERE gene in a neonate with type IV LTEC and type III esophageal atresia. Genetic alterations can contribute to the development of these congenital malformations; moreover, the role of environmental factors should not be excluded.

Subjects with terminal and interstitial deletions of chromosome 1p36 have a spectrum of defects that include eye anomalies, postnatal growth deficiency, structural brain anomalies, seizures, cognitive impairment, delayed motor development, behavior problems, hearing loss, cardiovascular malformations, cardiomyopathy, and renal anomalies [9, 16]. The proximal 1p36 genes that contribute to these defects have not been clearly delineated. RERE is located in the proximal region of chromosome 1p36. Due to its role as a nuclear receptor coregulator and the role it plays in retinoic acid signaling, it is considered a candidate gene, which could contribute to the development of several phenotypes seen in individuals with proximal interstitial deletions or large terminal deletions of 1p36 [9].

The function of RERE in development has been explored using mouse models. In these models, RERE plays a critical role in the development and function of multiple organs including the eye, brain, inner ear, heart, and kidney. To date, mutations in RERE have not been implicated as the cause of a specific disease or syndrome in humans; however, in 16% and 13% of the individuals with isolated 1p36 deletions that include RERE, orofacial clefts and genitourinary anomalies have, respectively, been reported [9].

The estimated annual incidence of LTEC is 1/10,000 to 1/20,000 live births, accounting for 0.2% to 1.5% of congenital malformations of the larynx. It is often associated with other congenital abnormalities (16% to 68%), mostly malformations of the digestive tract [7]. Our neonate presented with LTEC associated with esophageal atresia. There are scattered reports of patients with this association, but the actual incidence of this particular association is difficult to assess because most of the reports regarding this association are in the form of case reports or limited series [17–21].

It has been hypothesized that LTECs result from the complex interplay of multiple genes and environmental factors. In our patient, in whom LTEC and EA were also associated with a renal anomaly, the paternal origin of the genetic rearrangement was revealed. Even though the father had a normal phenotype, the causal role of RERE in malformations is supported. Indeed, the deletions in RERE may contribute alone or in conjunction with other genetic or environmental factors to the development of the phenotype seen in affected subjects [10]. The role of maternal obesity as a candidate cofactor is also proposed. The effects of maternal obesity extend to the fetus, with several large population-based analyses demonstrating independent risks of fetal neural tube defects, cardiac malformations, and orofacial clefts. The mechanism for the observed association between obesity and birth defects is not known, but several possible explanations have been proposed [22]. Firstly, obese women have metabolic alterations, such as hyperglycemia/diabetes or elevated insulin or estrogen levels that increase the risk for birth defects. Secondly, women who are obese also might have nutritional deficits, resulting from dieting behaviors or poor-quality diets that increase their risk for congenital anomalies. Additionally, obese women might have an increased requirement for certain nutrients (e.g., folic acid) known to be protective against birth defects. Associations between maternal obesity, epigenetic alterations, and congenital malformations have also been proposed [23, 24]. Finally, also considering the father's severe obesity, the role of the epigenetic paternal profile should not be excluded [25].

The surgical and anesthesiological management of patients affected by LTEC is generally complicated and presents a challenge in the preoperative, perioperative, and postoperative periods [5]. Recent advances in knowledge, diagnosis, and, above all, the treatment of LTEC have led to significant improvements in survival and quality of life for these patients, as obtained in this reported patient. As reported by Chitkara et al., critical factors in the successful management of these patients include a team-oriented approach with experience in airway surgery, safe management of the airway, early and aggressive management of gastric reflux, nutritional sustenance, and early surgical intervention [5].

In summary, we describe a neonate affected by type IV LTEC and type III esophageal atresia, in which a 1p36 deletion containing the RERE gene was detected and a multiteam approach resulted in successful surgical treatment. The causal role of the genetic profile was proposed; moreover, the fetal effects of concurrent parental factors should be considered. Future studies are mandatory to elucidate genomic and epigenomic susceptibility factors, which underlie these congenital malformations.
